# Supplementary material for: Dual-Energy Computed Tomography (DECT) for Diagnosing Contrast-Induced Encephalopathy (CIE) Mimicking Intracranial Hemorrhage (ICH): A Rare Case
Source: Diagnostics (Basel). 2025 Sep 23;15(19):2426. doi: 10.3390/diagnostics15192426 (PMC12523876; doi:10.3390/diagnostics15192426)
Supplement: Supplementary file 1 [file diagnostics-15-02426-s001.zip › diagnostics-3846248-supplementary.pdf]

Table S1. Quantitative DECT Parameters in Lesion vs. Contralateral Normal Tissue

	Lesion 1	contralateral normal tissue 1	Lesion 2	contralateral normal tissue 2
CT value (HU)	75	31	78	38
CT value of VNC map (HU)	27.9	30.7	34.4	32.0
IC map (mg/ml)	2.0	0.3	1.9	0.5
Zeff map	8.46	7.56	8.51	7.76

CT: computed tomography; DECT: dual-energy CT; HU: Hounsfield unit; VNC: virtual non-contrast; IC: iodine concentration; Zeff: effective atomic number.
